# Interaction between Red Yeast Rice and CYP450 Enzymes/P-Glycoprotein and Its Implication for the Clinical Pharmacokinetics of Lovastatin

**DOI:** 10.1155/2012/127043

**Published:** 2012-11-14

**Authors:** Chia-Hao Chen, Yow-Shieng Uang, Shang-Ta Wang, Jyh-Chin Yang, Chun-Jung Lin

**Affiliations:** ^1^School of Pharmacy, College of Medicine, National Taiwan University, Taipei 100, Taiwan; ^2^School of Pharmacy, Taipei Medical University, Taipei 100, Taiwan; ^3^Department of Internal Medicine, Hospital and College of Medicine, National Taiwan University, Taipei 100, Taiwan

## Abstract

Red yeast rice (RYR) can reduce cholesterol through its active component, lovastatin. This study was to investigate the pharmacokinetic properties of lovastatin in RYR products and potential RYR-drug interactions. Extracts of three registered RYR products (LipoCol Forte, Cholestin, and Xuezhikang) were more effective than pure lovastatin in inhibiting the activities of cytochrome P450 enzymes and P-glycoprotein. Among CYP450 enzymes, RYR showed the highest inhibition on CYP1A2 and CYP2C19, with comparable inhibitory potencies to the corresponding typical inhibitors. In healthy volunteers taking the RYR product LipoCol Forte, the pharmacokinetic properties of lovastatin and lovastatin acid were linear in the dose range of 1 to 4 capsules taken as a single dose and no significant accumulation was observed after multiple dosing. Concomitant use of one LipoCol Forte capsule with nifedipine did not change the pharmacokinetics of nifedipine. Yet, concomitant use of gemfibrozil with LipoCol Forte resulted in a significant increase in the plasma concentration of lovastatin acid. These findings suggest that the use of RYR products may not have effects on the pharmacokinetics of concomitant comedications despite their effects to inhibit the activities of CYP450 enzymes and P-gp, whereas gemfibrozil affects the pharmacokinetics of lovastatin acid when used concomitantly with RYR products.

## 1. Introduction

Cardiovascular disease remains the leading cause of morbidity and mortality worldwide and hyperlipidemia is a major contributing factor to the development of cardiovascular disease [1]. Red yeast rice (RYR), a fermented rice product generally produced using a specific strain of red yeast called *Monascus purpureus*, has been used as a medicinal food supplement to improve blood circulation by decreasing cholesterol levels [2–7]. The active component of RYR is generally considered to be monacolin K [8], also known as lovastatin, the compound equivalent to the statin drug Mevacor (Merck & Co., Inc., USA). Nonetheless, in addition to lovastatin, components of RYR products may include unsaturated fatty acids, sterols, amino acids, isoflavones, alkaloid, and trace element [2, 3, 9]. Interestingly, RYR products containing about 2–5 mg of lovastatin have a comparable efficacy to 20 mg of lovastatin in lowering blood cholesterol [2, 10–12], suggesting additive and/or synergistic pharmacological effects of RYR components.

Statins are a class of drugs that inhibit 3-hydroxy-3-methylglutaryl-coenzyme A reductase, an enzyme that plays a central role in the production of cholesterol in the liver [13]. Lovastatin is a prodrug that is converted by esterase to its active form, lovastatin acid. Lovastatin is also metabolized by CYP3A4 [14] and is a substrate of P-glycoprotein (P-gp) [15]. Because of its lovastatin content, the original Cholestin, a RYR product, is now considered as a drug by the US Food and Drug Administration (FDA), which also banned the sale of any dietary supplements containing Xuezhikang, a concentrated form of Cholestin. A new formulation of Cholestin that does not contain any RYR products is now sold in the USA, but the original formulation is still available as a RYR food supplement in Asia, New Zealand, and South Africa, while Xuezhikang is sold as dietary supplement in China and is now in phase 2 clinical trials in the USA. In addition, LipoCol Forte, with similar constituents to Xuezhikang, is a newly developed RYR product that is approved as a drug in Taiwan.

Although RYR products have been demonstrated to be well tolerated [10, 11, 16, 17], consumption of RYR products has been associated with occurrence of myopathy, rhabdomyolysis, or hepatitis in several case reports [18–21]. Nonetheless, it is noted that the occurrence of RYR-associated adverse reaction has usually been reported in patients with concomitant comedication [18–20]. However, due to limited information, most potential drug-RYR product interactions are inferred from known drug-statin interactions despite that RYR is not only a lovastatin-containing product. In this study, firstly, we investigated the in vitro inhibitory potencies of Cholestin, Xuezhikang, and LipoCol Forte on the activities of CYP450 enzymes and P-gp, compared to those of pure lovastatin. Secondly, the pharmacokinetic properties of lovastatin and its active metabolite, lovastatin acid, were evaluated in healthy volunteers taking the RYR product LipoCol Forte with or without concomitant use of nifedipine or gemfibrozil.

## 2. Methods

### 2.1. Materials

LipoCol Forte was purchased from NatureWise Biotech & Medicals Corporation, Taiwan, Xuezhikang from Peking University WBL Biotech Co., Ltd., China, and Cholestin from Nu Skin Pharmanex, USA. Nifedipine capsule (Adalat) was purchased from Bayer AG, Germany. Gemfibrozil capsule (Lopid) was purchased from Pfizer Inc., USA. Pure lovastatin was obtained from Merck KGaA (Darmstadt, Germany). Phenacetin, acetaminophen, tolbutamide, dextromethorphan, dextrorphan, 6*β*-hydroxytestosterone, furafylline, ticlopidine, sulfaphenazole, quinidine, and ketoconazole were from Sigma-Aldrich (M., USA). Bupropion, hydroxybupropion, S-mephenytoin, S-4-hydroxymephenytoin, and hydroxytolbutamide were from Toronto Research Chemicals Inc. (ON, Canada). Testosterone was from ACROS Organic (NJ, USA). Human liver microsomes (UltraPool HLM 150) were purchased from BD Biosciences (MA, USA). Other reagents and solvents were obtained from standard sources and were of the highest quality available.

### 2.2. Extraction Procedure for RYR Products

The ingredients of the RYR products were weighed and 600 mg extracted with 10 mL of methanol at room temperature. Following centrifugation (2,800 g for 10 min at room temperature), the supernatants were collected and evaporated to dryness under nitrogen. The analytical condition was performed according to the methods described previously with minor modifications [22]. To measure the lovastatin content, the residue was dissolved in methanol and lovastatin measured on a HPLC system (Hitachi, Japan) with a Biosil ODS column (4.6 mm × 150 mm, 5 *μ*m, Biotic Chemical Co., Ltd., Taiwan) with elution with a 70/30 v/v mixture of acetonitrile and 0.2% formic acid at a flow rate of 1.0 mL/min. Lovastatin was detected at 238 nm. For in vitro studies, the residue was dissolved in the appropriate assay buffer containing 1% methanol.

### 2.3. Inhibition of Cytochrome P450 (CYP) Enzyme Activity by RYR Product Extracts

The inhibition assay was performed according to the methods described previously with minor modifications [23, 24]. The activities of CYP450 enzymes (CYP1A2, CYP2B6, CYP2C9, CYP2C19, CYP2D6, and CYP3A4) in human liver microsomes were calculated from the ratios of typical substrates and their metabolites. Accordingly, the 6*β*-hydroxytestosterone/testosterone ratio was measured for CYP3A4, the 4′-hydroxymephenytoin/mephenytoin ratio for CYP2C19, the hydroxybupropion/bupropion ratio for CYP2B6, the hydroxytolbutamide/tolbutamide ratio for CYP2C9, the dextrorphan/dextromethorphan ratio for CYP2D6, and the acetaminophen/phenacetin ratio for CYP1A2. RYR product extracts or pure lovastatin at the same molar dose of lovastatin or CYP450 enzyme inhibitors (i.e., ketoconazole for CYP3A4, ticlopidine for CYP2C19 and 2B6, sulfaphenazole for CYP2C9, quinidine for CYP2D6, and furafylline for CYP1A2) were used to inhibit the metabolism of typical substrates of CYP450 enzymes. To measure inhibition of CYP enzyme activity, the reaction mixture contained 20 *μ*L of human liver microsomes (5 mg protein/mL in 500 mM potassium phosphate), 20 *μ*L of an NADPH-generating system (2.5 mM *β*-NADP, 25 mM G6P, 25 mM magnesium chloride, and 5 units/mL of G6PD), 20 *μ*L of 250 *μ*M marker substrates, and 0.5–25 *μ*M lovastatin (RYR product extracts or pure lovastatin) or 0.001–50 *μ*M specific inhibitor in a total volume of 100 *μ*L. After incubation at 37°C for 10 min (for dextromethorphan or testosterone), 20 min (for phenacetin or bupropion), 30 min (for tolbutamide), or 60 min (for S-mephenytoin), the reaction was terminated by addition of 50 *μ*L of acetonitrile and centrifugation at 9,000 g at 4°C for 15 min. An aliquot of 10 *μ*L of the supernatant was injected onto the LC/MS/MS system (Waters, MA, USA) (Section 2.7). The IC_50_ values for the RYR product extracts were determined from the relationship between the inhibitor concentration (*I*) and the percentage of metabolic activities at *I* by nonlinear regression analysis using Scientist v2.01 (MicroMath Scientific Software, Salt Lake City, UT, USA).

### 2.4. Inhibition of P-gp Activity by RYR Extracts

MDCK-MDR1 cells (2.5 × 10^4^ cells/cm^2^) (from The Netherlands Cancer Institute) were cultured in polystyrene Nunclon multidishes under an atmosphere of 5% CO_2_/95% air. The minimal essential medium (MEM) (Invitrogen, CA, USA) contained 10% fetal bovine serum (Hyclone, UT, USA), 5.5 mM L-glutamine, 1.5 mg/mL of sodium bicarbonate, and 1 mM sodium pyruvate (all from Sigma, MO, USA), and 100 unit/mL of penicillin and 0.1 mg/mL of streptomycin (both from Invitrogen). After 4 days, the cells were washed with ECF buffer (122 mM NaCl, 3 mM KCl, 25 mM NaHCO_3_, 1.2 mM MgSO_4_, 1.4 mM CaCl_2_, 10 mM D-glucose, 0.4 mM K_2_HPO_4_, and 10 mM HEPES, pH 7.4) and preincubated in ECF buffer for 30 min at 37°C, then digoxin uptake was initiated by addition of 40 nM ^3^H-digoxin (PerkinElmer Life Sciences Inc., MA, USA) in ECF buffer with or without extracts of RYR products containing 25 *μ*M lovastatin, 25 *μ*M pure lovastatin, or 25 *μ*M PSC833 (for IC_50_ studies, the concentrations used were 0.2–25 *μ*M) and the mixture incubated at 37°C for 60 min, then the reaction was stopped by aspirating the supernatant and adding 500 *μ*L of ice-cold ECF buffer, then the cells were solubilized overnight at room temperature in 1% Triton X-100 and radioactivity was measured by liquid scintillation counter using an external standard method for quench correction. The percentage of inhibition of P-gp activity and the IC_50_ values for P-gp inhibition were determined by the methods described by Rautio et al. [25].

### 2.5. Transcellular Transport Studies Across Caco-2 Cells

Caco-2 cells (1.0 × 10^5^ cells/cm^2^) were seeded into 30 mm Millicell inserts (Millipore, USA) and cultured according to the conditions described previously [26]. For transcellular transport studies, each insert chamber was filled with 1.5 mL of Hank's balanced salt solution containing 25 *μ*M lovastatin or the equivalent concentration of RYR product extract in the apical compartment, then after 30, 60, or 120 min incubation, a 250 *μ*L sample was collected from the basolateral compartment. At the end of the 120 min incubation, the cells were extracted with 1 mL of methanol by sonication for 30 min at room temperature and centrifuged at 14,000 g for 10 min at 4°C and the supernatants evaporated to dryness under nitrogen. The residues were reconstituted in 250 *μ*L of mobile phase and injected into the HPLC system described in the previous section (see extraction procedure for RYR products). 

### 2.6. Clinical Studies

#### 2.6.1. Subjects

All studies were approved by an institutional review board/ethics committee before initiation. The studies were registered at http://www.ClinicalTrials.gov/ (NCT01346657, NCT01269762, and NCT01385020) and were performed in accordance with the Declaration of Helsinki and according to good clinical practice guidelines and any relevant local laws, regulations, and guidelines. The subjects were 20 to 30 years old and in good health as determined by past medical history, physical examination, electrocardiogram, chest X-ray, laboratory tests, and urinalysis. Premenopausal women were required to have a negative pregnancy test. All subjects had to sign an informed consent form before being included in the study. The major exclusion criteria included clinical significant abnormalities detected on physical examination with the potential to alter the absorption or elimination of the study drugs or that could constitute a risk to the subject when taking the study drugs. In addition, subjects were excluded if they had taken any prescription or over-the-counter medications within 14 days before administration of the study drugs. 

#### 2.6.2. Single Dose Escalation and Multiple Dosing Studies

In the single dose study, the volunteers (*n* = 14) received one, two, or four LipoCol Forte capsules in the fed state in periods one to three, respectively, with a minimum of a 5-day washout period between each treatment. A high fat breakfast (including two eggs fried in butter, two strips of bacon, two slices of toast with butter, four ounces of hash brown potatoes, and eight ounces of whole milk) was provided 30 min before dosing. All doses were administered with 240 mL of water. Blood samples were collected prior to drug administration and at 0.5, 1, 1.5, 2, 2.5, 3, 4, 6, 8, and 12 h after taking the capsules. In the multiple dose study, the volunteers (*n* = 14) received one LipoCol Forte capsule in the fed state twice daily for 5 days. A high fat breakfast or dinner was served 30 min before dosing. One capsule was administered with 240 mL of water. Blood samples were drawn before and at 0.5, 1, 1.5, 2, 2.5, 3, 4, 6, 8, and 12 h after taking the ninth dose using vacutainers containing heparin and sodium fluoride. Within 30 min of collection, the samples were centrifuged at 2,600 g for 10 minutes at 4°C and the plasma transferred to labeled polypropylene tubes containing 50 *μ*L of 17% phosphoric acid and stored at −80°C until analysis.

#### 2.6.3. Interaction between Nifedipine and LipoCol Forte

Healthy subjects (*n* = 14) were randomly allocated to receive a single dose of either one 5 mg nifedipine capsule or one 5 mg nifedipine capsule plus one capsule of LipoCol Forte; after a washout of 7 days, the subjects received a single dose of the other drug. The subjects were fasted at least 10 h before dosing. The investigational products were administered with 240 mL of water. Blood samples were collected prior to the drug administration and at 0.167, 0.333, 0.5, 0.75, 1, 1.25, 1.5, 2, 2.5, 3, 4, 6, 8, 12, 14, 16, and 24 h after dosing using photophobic vacutainers containing heparin. Within 30 minutes of collection, samples were centrifuged at 2,600 g for 10 minutes at 4°C and the plasma transferred to labeled photophobic polypropylene tubes and stored at −80°C.

#### 2.6.4. Interaction between Gemfibrozil and LipoCol Forte

The volunteers (*n* = 13) took one capsule of LipoCol Forte orally with 240 mL water at 8 a.m.; blood samples were collected before and at 0.5, 1, 1.5, 2, 2.5, 3, 4, 6, 8, and 12 h after administration of LipoCol Forte. After a washout of 4 days, the subjects took gemfibrozil (two 300 mg Lopid capsules) with 240 mL water at 7 AM and 7 PM for 2 days, then, on the third day, took two gemfibrozil capsules at 7 AM and one LipoCol Forte capsule with 240 mL water at 8 AM and blood samples were collected as above. The volunteers were fasted overnight before dosing and a high fat breakfast or dinner was served 0.5 h after administration of gemfibrozil. In addition, a high fat breakfast was served 0.5 h before, and a meal was served 4 and 10 h after administration of LipoCol Forte. Blood samples were prepared as described in the previous section (dose-escalation study).

### 2.7. Sample Analysis

For the analysis of CYP450 enzyme markers, chromatographic separation carried out on a Biosil ODS column (4.6 mm × 150 mm, 5 *μ*m) with a mobile phase composed of acetonitrile (A) and 0.2% formic acid (B) using a gradient of 40%–75% (v/v) of solvent B at a flow rate of 1.0 mL/min with a postcolumn split volume ratio of 2/10 to the mass detector. To measure the metabolic activity of each CYP isoenzyme, the following marker substrates were quantified by running ESI^+^ MRM or ESI^−^ MRM analyses: the peak areas of m/z 180 → 110 for phenacetin, m/z 152 → 110 for acetaminophen, m/z 240 → 184 for bupropion, m/z 256 → 238 for hydroxybupropion, m/z 269 → 170 for tolbutamide, m/z 285 → 186 for hydroxytolbutamide, m/z 217 → 188 for (S)-mephenytoin, m/z 233 → 190 for (S)-4-hydroxymephenytoin, m/z 272 → 171 for dextromethorphan, m/z 258 → 157 for dextrorphan, m/z 289 → 97 for testosterone, and m/z 305 → 269 for 6*β*-hydroxytestosterone.

For the analysis of lovastatin and lovastatin acid in plasma, plasma samples (0.5 mL) were acidified with 0.2 M potassium dihydrogen phosphate and extracted with 4 mL of diethylether containing an internal standard (diclofenac in a final concentration of 50 ng/mL). After centrifugation, the diethylether layer was transferred to a clean tube and evaporated to dryness at ambient temperature under nitrogen and the residue reconstituted with 200 *μ*L of 75% acetonitrile containing 0.1% acetic acid and an aliquot (50 *μ*L) injected onto the LC/MS/MS system and separation was performed on a Biosil ODS column (4.6 mm × 150 mm, 5 *μ*m) with a mobile phase of acetonitrile/0.2% acetic acid (81/19) at a flow rate of 1.0 mL/min. All quantitative analyses were performed in ESI^+^ MRM mode: the peak areas of m/z 406 → 286 for lovastatin, m/z 423 → 303 for lovastatin acid, and m/z 296 → 215 for diclofenac.

For the analysis of nifedipine in plasma, plasma samples (0.2 mL) were extracted with 0.4 mL of acetonitrile containing an internal standard (atorvastatin in a final concentration of 20 ng/mL). After centrifugation, the supernatant (50 *μ*L) was injected onto the LC/MS/MS system and separation was performed on a Biosil ODS column (4.6 mm × 150 mm, 5 *μ*m) with a mobile phase of acetonitrile/1% formic acid (70/30) at a flow rate of 1.0 mL/min. All quantitative analyses were performed in ESI^+^ MRM mode: the peak areas of m/z 347 → 315 for nifedipine and m/z 559 → 440 for atorvastatin.

### 2.8. Pharmacokinetic Calculations

The plasma concentration-time data were used to calculate the pharmacokinetic parameters of lovastatin, lovastatin acid, and nifedipine by the noncompartment method using WinNonlin (version 5.2, Pharsight, USA). The pharmacokinetics were characterized, when appropriate, by the peak concentration in the plasma (*C*_max⁡_), time to reach the peak concentration (*T*_max⁡_), area under the plasma concentration versus time curve from time zero to the time of the last quantifiable concentration (AUC_0−*t*_), AUC from time zero to the time extrapolated to infinity (AUC_0−inf⁡_), half-life (*T*_1/2_), mean residence time (MRT), apparent clearance (CL/*F*), and apparent volume of distribution (*V*_*d*_/*F*). 

### 2.9. Statistical Analysis

Statistical analyses were performed using SYSTAT v12 (SYSTAT Software, Inc., Chicago, IL, USA). Statistical differences were evaluated by analysis of variance, with a level of significance of 0.05. Pairwise comparisons between groups were made using Fisher's least-significant difference test.

## 3. Results

### 3.1. Inhibitory Potencies of RYR Extracts on CYP450 Enzyme Activities

The amounts (mg/capsule) of lovastatin in the RYR products LipoCol Forte, Xuezhikang, and Cholestin were measured (Table 1). Methanol extracts of LipoCol Forte, Xuezhikang, and Cholestin and pure lovastatin (Merck) were adjusted to similar lovastatin concentrations in the appropriate assay buffer containing 1% methanol and their inhibitory potencies on the activities of CYP1A2, CYP2B6, CYP2C9, CYP2C19, CYP2D6, and CYP3A4 in human liver microsomes measured. As shown in Table 2, pure lovastatin inhibited the activity of CYP2B6, CYP2C9, CYP2C19, and CYP3A4 with IC_50_ values of about 9–16 *μ*M, but was less effective (IC_50_ > 50 *μ*M) on CYP1A2 and CYP2D6. In contrast, the three extracts from LipoCol Forte, Xuezhikang, and Cholestin exhibited higher inhibitory potencies for all six CYP450 enzymes, with IC_50_ values of about 1–8 *μ*M. In particular, the three extracts had comparable inhibitory potencies to typical inhibitors of CYP1A2 and CYP2C19, but were less effective than other CYP inhibitors.

### 3.2. Effects of RYR Extracts on P-gp Activities and Intestinal Permeability

**Figure 1:**
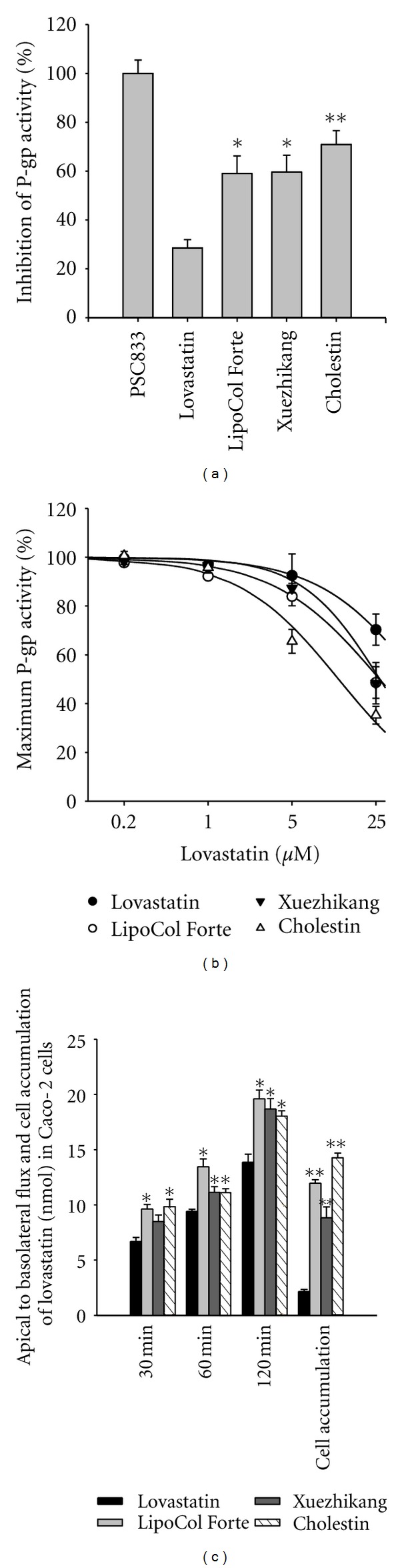
(a) Effects of 25 *μ*M pure lovastatin, extracts of RYR products containing 25 *μ*M lovastatin, or 25 *μ*M PSC833 on ^3^H-digoxin uptake by MDCK-MDR1 cells (*n* = 3–6). (b) ^3^H-digoxin uptake by MDCK-MDR1 cells in the presence of 0.2–25 *μ*M lovastatin or RYR product extracts at the same concentrations of lovastatin (*n* = 3). (c) Apical-to-basolateral flux and cellular accumulation of lovastatin after incubation of Caco-2 cells with 25 *μ*M lovastatin or the equivalent concentration of RYR product extract (*n* = 3). *P < 0.05 versus the lovastatin group; **P < 0.01 versus the lovastatin group.

The inhibitory effects of the RYR product extracts on P-gp was first investigated in MDCK-MDR1 cells using ^3^H-digoxin as substrate. PSC833, a P-gp inhibitor, at the concentration of 25 *μ*M resulted in the highest inhibition of digoxin uptake by MDCK-MDR1 cells, but the extracts of LipoCol Forte, Xuezhikang, and Cholestin (adjusted to 25 *μ*M lovastatin based on the content shown in Table 1) were also effective in inhibiting P-gp activity and significantly more effective than pure lovastatin (25 *μ*M) (Figure 1(a)). The inhibitory potencies on P-gp activity of different concentrations of lovastatin in LipoCol Forte, Xuezhikang, and Cholestin extracts were then measured in the same system. The IC_50_ values were estimated as 26.8 ± 12.4 *μ*M for LipoCol Forte, 27.0 ± 14.5 *μ*M for Xuezhikang, and 12.2 ± 3.0 *μ*M for Cholestin, all significantly lower than that of 56.4 ± 23.0 *μ*M for pure lovastatin (Figure 1(b)). The results using MDCK-MDR1 cells showed that the LipoCol Forte, Xuezhikang, and Cholestin extracts were potent inhibitors of P-gp in vitro. The effects of these RYR product extracts on the absorption of lovastatin were then examined in a model of intestinal absorption using cultured Caco-2 cells. As shown in Figure 1(c), apical-to-basolateral transport of lovastatin was significantly higher using extracts of LipoCol Forte, Xuezhikang, and Cholestin than using pure lovastatin at the same dose (25 *μ*M) of lovastatin, showing higher absorption of lovastatin in the presence of the RYR product extracts.

### 3.3. Single Dose Escalation and Multiple Dosing Studies of LipoCol Forte

Given the findings of these preclinical studies, LipoCol Forte was chosen for a clinical pharmacokinetic study, as it is an approved drug in Taiwan. To examine the dose linearity and time-dependent pharmacokinetics of a RYR product, LipoCol Forte was used in a single dose escalation study (1, 2, or 4 capsules of LipoCol Forte) and a multiple dose study (1 LipoCol Forte capsule twice daily for 5 days). In the single dose study, the AUC and *C*_max⁡_ for lovastatin and lovastatin acid increased proportionally with the dose of LipoCol Forte (Table 3), showing that the pharmacokinetic properties of lovastatin and lovastatin acid were linear within this dose range. When LipoCol Forte was given twice daily for consecutive 5 days, the pharmacokinetic properties remained unchanged (Table 3), showing that no accumulation of lovastatin or lovastatin acid and no enzyme induction/inhibition occurred with multiple dosing.

### 3.4. Interaction between Nifedipine and LipoCol Forte

Both lovastatin and the dihydropyridine calcium antagonist nifedipine are primarily metabolized by CYP3A4 [27]. Both drugs are concomitantly used in the therapy of hyperlipidemia and hypertension [28]. Therefore, the potential drug-drug interaction between nifedipine and LipoCol Forte was evaluated in this study. As shown in Table 4, the plasma concentration profile and pharmacokinetic parameters of nifedipine were not significantly changed with or without concomitant use of LipoCol Forte, indicating that LipoCol Forte had no effects on the pharmacokinetic properties of nifedipine.

### 3.5. Interaction between Gemfibrozil and LipoCol Forte

**Figure 2:**
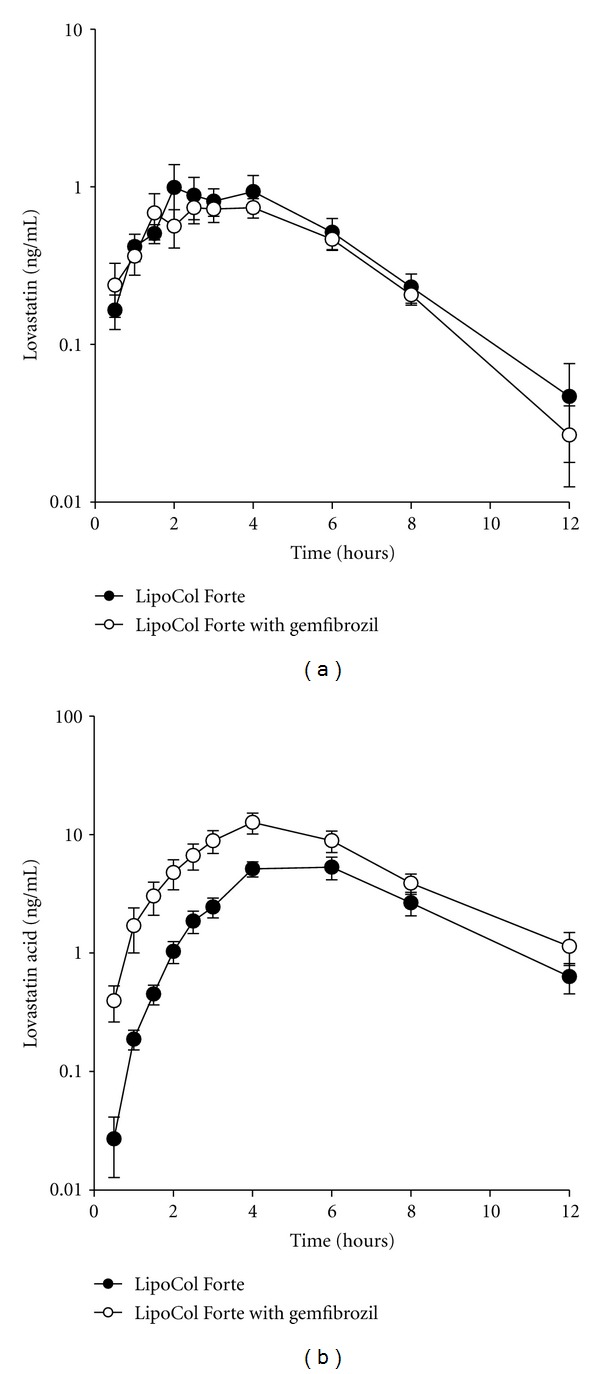
Plasma concentrations of lovastatin (a) and lovastatin acid (b) in healthy subjects after a single dose of one LipoCol Forte capsule alone or coadministration of gemfibrozil. The data are the mean ± SEM for 13 subjects.

A combination of a fibrate and a statin is effective in patients with mixed lipid disorders. Since an interaction between the fibrate gemfibrozil and lovastatin has been reported [29], possible drug-drug interaction was investigated between gemfibrozil and LipoCol Forte. As shown in Figure 2 and Table 5, the concomitant use of gemfibrozil had no significant effects on the pharmacokinetic parameters of lovastatin in LipoCol Forte, but the AUC and *C*_max⁡_ for lovastatin acid were significantly increased.

## 4. Discussion

Despite the fact that RYR products contain lovastatin and, in some countries, can be classified as drugs, in Asia, they are still considered as a food or dietary supplement and are widely available to the public. A number of studies have demonstrated the cholesterol lowering efficacy of RYR products [2–7], which were generally demonstrated to be well tolerated, with few safety concerns [10, 11, 16, 17]. However, consumption of RYR products has been associated with occurrence of myopathy, rhabdomyolysis, or hepatitis in several case reports [18–21]. Although RYR contains many components, it is generally believed that lovastatin is mainly responsible for the efficacy of RYR products. However, there is little information on the pharmacokinetic properties of lovastatin in these products. In this study, we first showed that extracts of different RYR products at the same lovastatin concentration differed in their ability to inhibit CYP450 enzymes and P-gp and LipoCol Forte, Xuezhikang, and Cholestin extracts at the same lovastatin content were more effective in inhibiting CYP3A4 and/or P-gp than pure lovastatin. It is also noted that extracts of LipoCol Forte and Xuezhikang had comparable inhibitory potencies to typical inhibitors of CYP2C19 and CYP1A2, suggesting their potential interaction with drugs that are primarily metabolized by these enzymes.

Lovastatin is metabolized by CYP3A4 [14] and is a substrate of P-glycoprotein (P-gp) [15]. The concomitant use of potent inhibitors of CYP3A4 or P-gp with lovastatin can significantly increase systemic exposure of lovastatin and lovastatin acid [30, 31]. Despite the observation that RYR product extracts could inhibit CYP450 enzymes (including CYP3A4) and P-gp in vitro, the pharmacokinetic properties in healthy volunteers of lovastatin and its active metabolite, lovastatin acid, were found to be linear in the dose range of 1 to 4 LipoCol Forte capsules taken as a single dose and no significant accumulation was observed after multiple dosing. Thus, its pharmacokinetic properties in healthy volunteers are predictable within the recommended dose range. 

It is suggested that the potential drug interactions can be evaluated in vitro by calculating the values of *I*_1_/IC_50_ or *I*_2_/IC_50_, in which *I*_1_ and *I*_2_ are the maximal plasma concentration at steady-state at the highest clinical dose and the oral molar concentration (oral dose divided by 250 mL) at the luminal side of gastrointestinal, respectively [32–34]. The average dosing of RYR products ranges from 600 to 1800 mg given twice daily [9, 11]. Despite the variation, the amount of lovastatin for each RYR dosing is about 5 mg, similar to one capsule (600 mg) of LipoCol Forte. In this regard, taking LipoCol Forte as an example, *I*_1_/IC_50_ values for RYR product to inhibit CYP3A4 and P-gp are about 0.001 and 0.0001 for CYP3A4 and p-gp, respectively. Both are below the suggested cut-off value of 0.1 [32, 34], suggesting no potential interaction. On the other hand, *I*_2_/IC_50_ values for RYR product to inhibit CYP3A4 and P-gp are 18 and 2.1, respectively. According to FDA guidance, this suggests the likelihood of in vivo interaction between RYR and CYP3A4 substrates, based on the *I*_2_/IC_50_ value of larger than 10 [33, 34]. Previous study suggests no significant interaction between lovastatin and nifedipine, a substrate of CYP3A4 [35]. However, our data suggest that RYR is more potent than lovastatin in inhibiting CYP3A4 and may interact with nifedipine in vivo. Accordingly, the effects of RYR (one 600 mg LipoCol Forte capsule) on the pharmacokinetic properties of nifedipine were investigated in healthy volunteers. The results showed that the concomitant use of LipoCol Forte had no effects on the pharmacokinetic properties of nifedipine, probably due to the marginal *I*_2_/IC_50_ ratio at this dose. Alternatively, solubility problem may lead to false-positive estimation utilizing the proposed *I*_2_/IC_50_ criteria [36, 37]. Nonetheless, the effects of RYR at higher doses need to be further confirmed.

Given that occurrence of RYR-associated adverse reactions has usually been reported in patients with concomitant comedications [18–21], possible drug-RYR product interactions need to be further addressed. Due to limited information, most potential drug-RYR product interactions are inferred from known drug-drug interactions for statins. Among these, an interaction between fibrates and statins has been noted by the US FDA [38]. Fibrates are carboxylic acids that are commonly used in conjunction with statins to treat hyperlipidemia and it has been observed that concomitant use of the fibrate gemfibrozil with Mevacor markedly increases plasma concentrations of the active lovastatin acid without affecting those of the parent lovastatin [29]. Likewise, in the present study, the concomitant use of gemfibrozil and LipoCol Forte increased plasma concentrations of lovastatin acid without affecting lovastatin levels. It has been demonstrated that lovastatin acid is metabolized by CYP2C8 [39], whereas gemfibrozil and its glucuronide metabolite are inhibitors of CYP2C8 [39]. Gemfibrozil may also inhibit the OATP1B1-mediated hepatic uptake of lovastatin acid, as it was reported for other statin acids [40]. These may be the reasons why gemfibrozil increased the AUC value of the active lovastatin acid but not of the parent lovastatin. 

A number of drugs, including fibrates, azole anti-fungals, macrolide antibiotics, anti-arrhythmics, and protease inhibitors, may increase the plasma levels of statins, through the inhibition of CYP3A4, P-gp, or OATP1B1 [30, 31]. Given that RYR is a lovastatin-containing drug, the concomitant use of these drugs with RYR should be cautious, as it was demonstrated in the gemfibrozil case in the present study. On the other hand, since RYR had comparable inhibitory potencies to typical inhibitors of CYP1A2 and CYP2C19, the interaction between RYR and substrates of CYP1A2 (e.g., verapamil) [41] and/or CYP2C19 (e.g., clopidogrel, a drug that is activated by both CYP1A2 and CYP2C19) [42, 43] is worth an attention. In particular, for CYP2C19, 13% to 23% of Asian populations are CYP2C19 poor metabolizers and the pharmacokinetics and pharmacodynamics of CYP2C19 substrates are dependent on CYP2C19 genotypes [44, 45]. In this regard, it is noteworthy to study the effects of RYR on the metabolism of CYP2C19 substrates in CYP2C19 poor metabolizers.

## 5. Conclusions

Pharmacokinetics of lovastatin and its active metabolite, lovastatin acid, are linear in the dose range of 1 to 4 LipoCol Forte capsules taken as a single dose and no significant accumulation was observed after multiple dosing. Extracts of RYR products (LipoCol Forte, Xuezhikang, and Cholestin) are more effective than pure lovastatin in inhibiting the activities of CYP450 enzymes and P-gp in vitro, whereas the use of one LipoCol Forte capsule does not change the pharmacokinetics of nifedipine in vivo. Nevertheless, the interaction between RYR products and substrates of CYP1A2 and CYP2C19 is worth an attention. On the other hand, the concomitant use of gemfibrozil with LipoCol Forte increases the plasma concentrations of lovastatin acid. It should be cautious when RYR products are used with drugs that can interact with lovastatin.

## Figures and Tables

**Table 1:** Lovastatin content of the different red yeast rice products.

	LipoCol Forte	Xuezhikang	Cholestin
Measured lovastatin content (mg/capsule)	5.35 ± 0.19	2.36 ± 0.21	0.96 ± 0.05
Lovastatin content according to the label (mg/capsule)	5.7	2.5	2.4
Error percentage	−6.14	−5.64	−60.1
Usual dose	One 600 mg capsule twice daily	Two 300 mg capsules twice daily	Two 600 mg capsules twice daily

The data are the mean ± SEM for the content per capsule for six separate preparations.

**Table 2:** Inhibitory potencies of extracts of RYR products or pure lovastatin on the activities of different CYP450 enzymes in human liver microsomes.

IC_50_ (*μ*M)	Pure lovastatin	LipoCol Forte	Xuezhikang	Cholestin	Specific CYP inhibitor*
CYP1A2	>100 *μ*M	5.21 ± 0.15^a^	2.45 ± 0.32^a,b ^	7.26 ± 0.39^a^	3.11 ± 0.36
CYP2B6	14.96 ± 1.11	2.49 ± 0.40^a^	3.48 ± 0.38^a^	5.45 ± 0.53^a^	0.48 ± 0.07
CYP2C9	16.87 ± 3.25	1.85 ± 0.17^a^	2.49 ± 0.10^a^	3.93 ± 0.35^a^	0.152 ± 0.001
CYP2C19	9.85 ± 1.20	1.52 ± 0.48^a,b^	2.67 ± 0.82^a,b^	1.62 ± 0.09^a,b^	2.49 ± 1.01
CYP2D6	>50 *μ*M	3.97 ± 1.33^a^	5.52 ± 0.98^a^	7.26 ± 2.70^a^	0.44 ± 0.19
CYP3A4	14.04 ± 1.21	3.13 ± 0.18^a^	1.72 ± 0.13^a^	8.26 ± 0.44^a^	0.062 ± 0.002

The data are the mean ± SEM for three to four separate preparations.

*The specific inhibitors were furafylline for CYP1A2, ticlopidine for CYP2B6 and CYP2C19, sulfaphenazole for CYP2C9, quinidine for CYP2D6, and ketoconazole for CYP3A4.

^a^ *P* value < 0.05 compared to pure lovastatin; ^b^ *P* value > 0.05 compared to the specific inhibitor for each enzyme.

**Table 3:** Pharmacokinetic parameters of lovastatin and lovastatin acid in healthy subjects after a single dose of 1, 2, or 4 LipoCol Forte capsules and multiple doses of one LipoCol Forte capsule twice daily for 5 days in the fed state. The data are the mean ± SD for 14 subjects.

	Single dose (one capsule)	Single dose (two capsules)	Single dose (four capsules)	Multiple doses (one capsule BID)
Lovastatin				
AUC_0–12_ (ng × h/mL)	6.74 ± 3.27*	13.38 ± 6.07*	24.95 ± 14.25*	5.22 ± 2.60
AUC_0–inf_ (ng × h/mL)	7.47 ± 3.44*	14.36 ± 6.30*	26.78 ± 15.36*	5.71 ± 2.68
*C*_max⁡_ (ng/mL)	1.61 ± 0.80*	3.36 ± 1.81*	5.70 ± 3.42*	1.41 ± 0.69
AUC_0–12_/dose	1.18 ± 0.57	1.17 ± 0.53	1.09 ± 0.63	0.92 ± 0.46
AUC_0–inf_/dose	1.31 ± 0.60	1.26 ± 0.55	1.17 ± 0.67	1.00 ± 0.47
*C*_max⁡_/dose	0.28 ± 0.14	0.30 ± 0.16	0.25 ± 0.15	0.25 ± 0.12
*T*_max⁡_ (h)	3.46 ± 1.28	3.29 ± 1.35	3.25 ± 0.78	3.39 ± 1.38
MRT (h)	5.55 ± 1.98	5.27 ± 1.42	5.51 ± 1.11	4.81 ± 1.24
*T*_1/2_ (h)	2.36 ± 0.66	2.36 ± 0.68	2.42 ± 0.45	1.93 ± 0.43
CL/*F* (L/h)	941.7 ± 453.5	994.5 ± 548.8	1128.8 ± 624.4	1339.5 ± 763.3
*V*_*d*_/*F* (L)	3078.4 ± 1327.7	3380.7 ± 2049.5	3862.9 ± 1956.5	3569.5 ± 1801.1
Lovastatin acid				
AUC_0–12_ (ng × h/mL)	33.0 ± 13.1*	79.1 ± 27.6*	158.3 ± 60.2*	32.4 ± 9.7
AUC_0–inf_ (ng × h/mL)	38.3 ± 16.2*	85.4 ± 27.8*	169.1 ± 62.9*	33.7 ± 9.7
*C*_max⁡_ (ng/mL)	7.78 ± 4.12*	17.80 ± 9.21*	36.24 ± 17.12*	8.04 ± 3.24
AUC_0–12_/dose	5.75 ± 2.30	6.94 ± 2.42	6.94 ± 2.64	5.69 ± 1.70
AUC_0–inf_/dose	6.72 ± 2.85	7.50 ± 2.44	7.42 ± 2.76	5.92 ± 1.70
*C*_max⁡_/dose	1.37 ± 0.72	1.56 ± 0.81	1.59 ± 0.75	1.41 ± 0.57
*T*_max⁡_ (h)	5.07 ± 1.59	5.14 ± 1.70	5.00 ± 1.52	4.36 ± 0.93
MRT (h)	7.15 ± 3.30	6.43 ± 1.77	6.39 ± 1.43	5.59 ± 1.15
*T*_1/2_ (h)	2.57 ± 1.74	2.07 ± 0.68	2.04 ± 0.68	1.92 ± 0.34
CL/*F* (L/h)	172.9 ± 68.1	145.7 ± 43.1	150.2 ± 45.9	192.3 ± 63.7
*V*_*d*_/*F* (L)	602.8 ± 336.8	438.4 ± 188.6	455.9 ± 265.2	544.1 ± 240.9

*P < 0.05 among the single dose of one, two, and four capsule groups.

**Table 4:** Pharmacokinetic parameters of nifedipine in healthy subjects after a single dose of 5 mg nifedipine capsule with or without 600 mg LipoCol Forte capsule in the fasted state. The data were given as mean ± SD for 14 subjects.

	Nifedipine alone	Nifedipine with LipoCol Forte
	Mean ± SD	Mean ± SD
AUC_0–16_ (ng ×h/mL)	131.7 ± 37.0	135.3 ± 27.8
AUC_0–inf_ (ng × h/mL)	136.5 ± 38.2	140.8 ± 29.4
*C*_max⁡_ (ng/mL)	81.46 ± 25.62	71.78 ± 23.56
*T*_max⁡_ (h)	0.46 ± 0.15	0.55 ± 0.43
MRT (h)	3.68 ± 0.59	3.89 ± 0.97
*T*_1/2_(h)	3.62 ± 0.90	3.58 ± 1.39
CL/*F* (L/h)	40.2 ± 14.9	37.3 ± 9.9
*V*_*d*_/*F* (L)	201.4 ± 56.1	186.8 ± 69.9

**Table 5:** Pharmacokinetic parameters of lovastatin and lovastatin acid in healthy subjects after a single dose of one LipoCol Forte capsule alone or with coadministration of 600 mg of gemfibrozil twice daily. The data are the mean ± SD for 13 subjects.

	LipoCol Forte	LipoCol Forte with gemfibrozil	LipoCol Forte	LipoCol Forte with gemfibrozil
	Lovastatin		Lovastatin acid	
AUC_0–12_ (ng × h/mL)	5.00 ± 3.29	4.25 ± 1.78	31.1 ± 16.5	65.5 ± 31.6*
AUC_0–inf_ (ng × h/mL)	5.68 ± 3.48	5.04 ± 1.86	33.1 ± 18.2	69.7 ± 31.7*
*C*_max⁡_ (ng/mL)	1.53 ± 1.45	1.21 ± 0.67	6.79 ± 3.78	14.31 ± 7.43*
*T*_max⁡_ (h)	3.08 ± 1.17	3.00 ± 1.37	4.85 ± 1.14	3.73 ± 1.20
MRT (h)	4.87 ± 1.28	5.32 ± 1.68	6.19 ± 1.38	5.77 ± 1.87
*T*_1/2_(h)	2.12 ± 0.70	2.55 ± 1.13	1.94 ± 0.75	1.94 ± 0.70
CL/*F* (L/h)	1374.0 ± 782.6	1372.5 ± 763.8	218.4 ± 108.6	99.9 ± 48.4*
*V*_*d*_/*F* (L)	4000.5 ± 2599.0	4512.9 ± 1805.5	644.3 ± 586.2	277.6 ± 154.8*

*P < 0.05 versus the treatment of LipoCol Forte capsule alone.
